# Book Review: A Computational View of Autism: Using Virtual Reality Technologies in Autism Intervention

**DOI:** 10.3389/fpsyg.2021.722672

**Published:** 2021-09-20

**Authors:** Sen Yang, Jing Zhang

**Affiliations:** ^1^School of Information Engineering, Hangzhou Vocational & Technical College, Hangzhou, China; ^2^Institute of Philosophy, Hangzhou Dianzi University, Hangzhou, China; ^3^School of Medicine, Technische Universität Dresden, Dresden, Germany

**Keywords:** autism spectrum disorders, autism intervention, virtual reality, robotic technology, computer-based technology, technology-assisted training

Autism spectrum disorder (ASD) is a generalized neurodevelopmental disorder whose main characteristics are summarized as persistent defects in social communication and social interaction in various situations or showing limited and repetitive behavior patterns, interests, or activities (APA, 2013). The number of diagnosed autism has increased significantly (Christensen et al., 2016). Clinical studies show that patients with ASD, especially children, are often accompanied by other symptoms, such as anxiety disorder, catatonic symptoms, and attention deficit hyperactivity disorder. These aspects seriously affect the learning and development of ASD individuals and bring endless troubles to their families. The traditional one-on-one, face-to-face interventions led by professional therapists have been proved to be effective, but problems, such as limited resources and high cost, are also prominent. Some researchers have turned to computer science for help (Goodwin, 2008).

In the book *A Computational View of Autism: Using Virtual Reality Technology in Autism Intervention*, based on her nearly 10-year experience applying technology to ASD research, Uttama Lahiri tries to illustrate how to integrate computer science and technology with autism intervention in a comprehensive and detailed way.

The book consists of six chapters. Chapter 1 (“Autism and Its Prevalence”) focuses on introducing frequently-used assessment methods. Seven scales/questionnaires are introduced. Lahiri explains each scale's constitution, its proper evaluation subjects, and the diagnostic criteria. A differential diagnosis of potential autistic individuals can be made with these scales through parents' or caregivers' feedback. Diagnosis is the first step for further intervention. Then, Chapter 2 (“Conventional Intervention Techniques”) exemplifies traditional approaches. In home-based settings, by describing five specific examples of applying social stories, Lahiri explains how to teach autistic children skills about sharing, initiating a conversation, acting appropriate table manners, and applying other common social skills. In community-based and school-based settings, the author mainly introduces Applied Behavior Analysis (ABA) therapy. ABA has been proved effective in overcoming the obstacles caused by the aberrant behavior of autistic children and helps improve their performance in imitation, conversation, and communication. The effect of traditional interventions is undeniable, but its shortcomings are also apparent. Fortunately, with computer science and technology development, performing technologies-based intervention among autism has become increasingly mature.

In Chapter 3 (“Role of Technology in Autism Intervention”), the author analyzes the feasibility of developing new intervention platforms through technology. Adopting technology in autism intervention can effectively avoid the shortcomings of traditional methods and even produce extra advantages. The technology-based intervention can integrate more functions, thus improving communication ability more efficiently, identify emotions more scientifically, and quantify the emotional state more accurately. Moreover, the technology-based interventions are excel in controllability, personalization, and scalability. Specifically, new interventions mainly rely on robotic and computer-based technology. The former is closer to reality but with a relatively higher cost, and the latter is more suitable for generalization but needs to be cautious about leading the patients further away from reality. One possible way to integrate robotic technology with computer-based technology is by adopting virtual reality (VR).

VR uses a series of technological means, such as monocular or stereoscopic display, tracking technologies, and augmented reality to integrate the virtual and real world. It involves interactive video tasks, a virtual environment, and a multisensory experience. Such particularity makes it equipped with unique advantages in dealing with mental health, especially for ASD (Fridhi et al., 2018). VR's dynamic and vivid social scene platform can highly simulate the natural environment, while more importantly, patients do not need to worry about mistakes and rejection in the virtual environment. Chapter 4 (“Scope of Virtual Reality to Autism Intervention”) illustrates immersive VR and augmented reality applications, respectively. The former is wildly used to teach shopping skills, navigation skills, and social communication skills, while the latter is often used to teach self-related aspects, pretend play skills, and non-verbal social communication skills.

There is no doubt that technology development has extensively promoted the diagnosis, intervention, and treatment of ASD. For more effective intervention, appropriate design is essential. In Chapter 5 (“Design of Virtual Reality-Based Applications in Autism”), Lahiri introduces matters that should be paid attention to when building VR-based scenes for different applications, such as the graphical user interface of stimuli, the adaptive characteristics, and the avatars of virtual characters. In the last chapter (“Technology-Assisted Skills Training: The Way Forward”), she predicts that using computational intelligence to build a training platform, carrying out autism intervention, and providing personalized skills training for autistic children will be fully realized shortly. Moreover, the technology-based intervention may further understand autistic children's unique world to better tap their potential.

However, it is crucial to avoid exaggerating the effectiveness of VR intervention. One systematic review reveals that most studies only analyze the improvement of ASD participants through VR intervention without sufficient contrastive analysis with either the subjects in the control group or the effects of other intervention methods (Mesa-Gresa et al., 2018). For example, the effectiveness of VR is not more significant than that of other technologies, such as video modeling (Fitzgerald et al., 2018). Additionally, the subjects of many studies are high-functioning; therefore, whether VR is suitable for all types of ASD needs more tests. At these points, this book overemphasizes the positive role of VR and ignores the rational analysis of its advantages and disadvantages to a certain extent. Besides, multisensory integration is probably a new hotspot for future directions. More and more studies have shown that ASD individuals show certain atypical interoceptive defects (Mul et al., 2018). The application of VR technology in multisensory integration and emotional arousal may contribute to the effectiveness of ASD intervention (Muszynski et al., 2021).

Still and all, this book gives a comprehensive presentation of ASD, including its characteristics, diagnostic criteria, traditional interventions, and technology-based interventions. It provides direct guidance for clinicians in designing interventions. Both non-specialists and professional therapists of ASD will benefit from reading this book.

## Author Contributions

SY and JZ wrote the manuscript, with larger contributions by SY. JZ then provided edits and suggestions for revision.

## Funding

This work was supported by The National Social Science Foundation (20FZXB017) and Fundamental Research Funds for the Provincial Universities of Zhejiang (GK219909299001-204).

## Conflict of Interest

The authors declare that the research was conducted in the absence of any commercial or financial relationships that could be construed as a potential conflict of interest.

## Publisher's Note

All claims expressed in this article are solely those of the authors and do not necessarily represent those of their affiliated organizations, or those of the publisher, the editors and the reviewers. Any product that may be evaluated in this article, or claim that may be made by its manufacturer, is not guaranteed or endorsed by the publisher.
